# Comparison of the Microbiological Quality and Safety between Conventional and Organic Vegetables Sold in Malaysia

**DOI:** 10.3389/fmicb.2017.01433

**Published:** 2017-07-31

**Authors:** Chee-Hao Kuan, Yaya Rukayadi, Siti H. Ahmad, Che W. J. Wan Mohamed Radzi, Tze-Young Thung, Jayasekara M. K. J. K. Premarathne, Wei-San Chang, Yuet-Ying Loo, Chia-Wanq Tan, Othman B. Ramzi, Siti N. Mohd Fadzil, Chee-Sian Kuan, Siok-Koon Yeo, Mitsuaki Nishibuchi, Son Radu

**Affiliations:** ^1^Department of Food Science, Faculty of Food Science and Technology, Universiti Putra Malaysia Serdang, Malaysia; ^2^Department of Crop Science, Faculty of Agriculture, Universiti Putra Malaysia Serdang, Malaysia; ^3^Department of Science and Technology Studies, Faculty of Science, University of Malaya Kuala Lumpur, Malaysia; ^4^Neogenix Laboratoire Sdn Bhd Petaling Jaya, Malaysia; ^5^School of Biosciences, Taylor's University Lakeside Subang Jaya, Malaysia; ^6^Center for Southeast Asian Studies, Kyoto University Kyoto, Japan; ^7^Food Safety and Food Integrity, Institute of Tropical Agriculture and Food Security, Universiti Putra Malaysia Seri Kembangan, Malaysia

**Keywords:** *Escherichia coli* O157:H7, salmonella, *Listeria monocytogenes*, fresh produce, organic farming

## Abstract

Given the remarkable increase of public interest in organic food products, it is indeed critical to evaluate the microbiological risk associated with consumption of fresh organic produce. Organic farming practices including the use of animal manures may increase the risk of microbiological contamination as manure can act as a vehicle for transmission of foodborne pathogens. This study aimed to determine and compare the microbiological status between organic and conventional fresh produce at the retail level in Malaysia. A total of 152 organic and conventional vegetables were purchased at retail markets in Malaysia. Samples were analyzed for mesophilic aerobic bacteria, yeasts and molds, and total coliforms using conventional microbiological methods. Combination methods of most probable number-multiplex polymerase chain reaction (MPN-mPCR) were used to detect and quantify foodborne pathogens, including *Escherichia coli* O157:H7, Shiga toxin-producing *E. coli* (STEC), *Listeria monocytogenes, Salmonella* Typhimurium, and *Salmonella* Enteritidis. Results indicated that most types of organic and conventional vegetables possessed similar microbial count (*P* > 0.05) of mesophilic aerobic bacteria, yeasts and molds, and total coliforms. *E. coli* O157:H7 and *S*. Typhimurium were not detected in any sample analyzed in this study. Among the 152 samples tested, only the conventional lettuce and organic carrot were tested positive for STEC and *S*. Enteritidis, respectively. *L. monocytogenes* were more frequently detected in both organic (9.1%) and conventional vegetables (2.7%) as compared to *E. coli* O157:H7, *S*. Typhimurium, and *S*. Enteritidis. Overall, no trend was shown that either organically or conventionally grown vegetables have posed greater microbiological risks. These findings indicated that one particular type of farming practices would not affect the microbiological profiles of fresh produce. Therefore, regardless of farming methods, all vegetables should be subjected to appropriate post-harvest handling practices from farm to fork to ensure the quality and safety of the fresh produce.

## Introduction

Public awareness of healthy eating habits have been intensified in recent years and prompted an increased demand for fresh fruits and vegetables (Olaimat and Holley, 2012). Despite the health benefits derived from consuming fresh produce, the risk of microbiological contamination in vegetables is of concern as the contamination can possibly occur through the food chain, from farm to fork. Over the past decade, numerous foodborne disease outbreaks caused by *Listeria monocytogenes, Escherichia coli* O157:H7, and *Salmonella* spp. were related to the consumption of contaminated fresh vegetables (Beuchat, 2002; Centers for Disease Control and Prevention, 2011, 2012; Maffei et al., 2013).

Given people's growing awareness of health and environmental sustainability, organic farming systems have become more well-received because conventional farming uses large amounts of synthetic pesticides and chemical fertilizers. Organic foods are perceived as safer and more healthful foods due to the chemical-free farming techniques used for their production as compared to conventionally produced foods (Somasundram et al., 2016). In Europe, the consumption of fresh organic produce has increased annually (Willer and Kilcher, 2009). In Malaysia, although the organic food remains a niche market and comprises only a small fraction of the food market, the demand for organic food has grown steadily. There were about 131 ha organic farms in Malaysia in 2001. However, the land area for organic farms was increased by 18-fold–2,367 ha, of which 962 ha are certified organic within a 5-year period. In Malaysia, the production of organic food is limited to vegetables and fruits only and most of the fresh organic produce are sold in domestic markets (Mohamad et al., 2014; Tiraieyari et al., 2014; Somasundram et al., 2016).

Despite the widespread consumers' belief that organic foods are “safer” and “more healthful” than conventional foods, evidence to support this concept is difficult to determine. Microbiological quality and safety of organic produce remain to be controversial and debated (Magkos et al., 2006). This issue emerged due to the lack of research and limited scientific data to reveal the actual scenario. The view that fresh organic produce is “safer” than conventionally grown food appears to be constructed on the perception that organic fruits and vegetables are free from chemical fertilizers and synthetic pesticides (Institute of Food Technologists, 2000; Somasundram et al., 2016). Conversely, previous studies have suggested that organic production practices, such as the use of manure may increase the risk of microbiological contamination. Manure may harbor foodborne pathogens, such as *Salmonella* spp., *L. monocytogenes*, and *E. coli* O157:H7 (Stephenson, 1997; McMahon and Wilson, 2001; Williams, 2002; Johannessen et al., 2004). Also, manure may introduce various pathogenic microorganisms that can persist for a long duration in the soil (Pell, 1997). However, it is difficult to conclude that the consumption of fresh organic produce would confer greater microbiological risk to consumers than conventional food. Other than cultivation method, microbial contamination can occur during harvesting, post-harvest handling or at any point along the food supply chain (Beuchat and Ryu, 1997).

The present study aimed to investigate and compare the microbiological status of different organic and conventional vegetables sold in the retail markets in Malaysia. To the best of our knowledge, this is the first comprehensive study on the comparison of microbiological quality and safety level between organically and conventionally grown vegetables in Southeast Asia.

## Materials and methods

### Sample collection

A total of 152 organic and conventional vegetables, comprising of 77 organic (certified by competent national and overseas authorities) and 75 conventional, were randomly purchased from hypermarkets and wet markets in Kuala Lumpur, Selangor, and Putrajaya. The samples collected included: cabbage (*Brassica oleracea*), carrot (*Daucus carota* subsp. *sativus*), calamondin (× *Citrofortunella microcarpa)*, cherry tomato (*Solanum lycopersicum* var. *cerasiforme*), Bird's eye chili (*Capsicum annum*), cucumber (*Cucumis sativus*), eggplant (*Solanum melongena*), winged bean (*Psophocarpus tetragonolobus*), Romaine lettuce (*Lactuca sativa* var. *longifolia*), Iceberg lettuce (*Lactuca sativa* var. *capitata*), *Looseleaf lettuce* (*Lactuca sativa* var. *crispa*), *Butterhead lettuce* (*Lactuca sativa* var. *capitata*), sweet potato (*Ipomoea batatas*), tomato (*Solanum lycopersicum*), and white radish (*Raphanus sativus*). Sampling was carried out over a 1-year period (November 2015 to October 2016). All samples (250–300 g each) were randomly collected from bulk quantities of vegetables, placed in sterile bags (BagMixer® 400 mL, Interscience, Saint-Nom-la-Bretèche, France), kept in an insulated box with ice packs and transported immediately to the Food Safety and Quality Laboratory, Universiti Putra Malaysia for microbiological analyses.

### Microbiological analysis

Twenty-five grams of each sample was cut into small pieces, weighed, placed in a sterile stomacher bag, and followed by homogenization using a stomacher machine (BagMixer® 400P, Interscience, Saint-Nom-la-Bretèche, France) with 225 mL of 0.1% (v/v) peptone water (Oxoid™, Basingstoke, Hampshire, UK) for 1 min. The pH of the bacterial culture broth was neutralized to pH 7.0 with 0.5 M NaOH solution. Mesophilic aerobic bacteria, total coliforms, and yeasts and molds were enumerated using conventional methods (Beuchat and Cousin, 2001; Kornacki and Johnson, 2001; Morton, 2001). Each sample was analyzed in triplicate and all the results were expressed as colony-forming units per gram (CFU/g).

### Detection and enumeration of foodborne pathogens by MPN-PCR method

#### Most-probable-number (MPN)

Each sample was cut into small pieces, and then a total of 10 g of sample was mixed with 90 mL of Tryptic Soy Broth (TSB; Merck, Darmstadt, Hesse, Germany), Listeria Enrichment Broth (LEB; Merck, Darmstadt, Hesse, Germany), and Buffered Peptone Water (BPW; Merck, Darmstadt, Hesse, Germany) for detection of *E. coli* O157:H7, *Listeria* spp., and *Salmonella* spp., respectively, in sterile stomacher bag and homogenized using a stomacher machine (BagMixer® 400P, Interscience, Saint-Nom-la-Bretèche, France) for 1 min. The pH of the enrichment broths was adjusted to pH 7.0 with 0.5 M NaOH solution before incubation. For the three-tube MPN analysis, 1 mL of the 10-, 100-, and 1,000-fold dilutions of the enriched bacteria culture were incubated in MPN tubes for 24 h at 37°C for detection and enumeration of *E. coli* O157:H7 and *Salmonella* spp., and 48 h at 30°C for detection and enumeration of *Listeria* species.

#### Genomic DNA extraction and multiplex-PCR

All the incubated MPN tubes were subjected to DNA extraction using the boiled-cell method as described in Table 1. Multiplex-PCR assays and gel electrophoresis for the detection of Shiga toxin-producing *E. coli* (STEC), *E. coli* O157:H7, *Listeria* spp., *L. monocytogenes, Salmonella* spp., *S*. Enteritidis, and *S*. Typhimurium were performed based on the methods as summarized in Table 2.

**Table 1:** Procedures for extraction of pathogens genomic DNA from conventional and organic vegetables.

**Target pathogens**	**Genomic DNA extraction procedures**	**References**
STEC O157:H7 and STEC non-O157	Each MPN tube was centrifuged at 13,400 × *g* for 1 min. The supernatants were discarded and the pellets were resuspended with 200 μL of Tris-EDTA buffer. The mixture was then boiled for 10 min and cooled at −20°C immediately for another 10 min before it was centrifuged at 13,400 × *g* for 2 min. The supernatant was used as a template DNA in PCR assays.	Loo et al., 2013
*Listeria* spp. and *L. monocytogenes*	Each MPN tube was centrifuged at 13,400 × *g* for 1 min. The supernatants were discarded and the pellets were resuspended in 200 μL of Tris-EDTA buffer. The mixture was then boiled for 10 min and cooled at −20°C immediately for another 10 min before it was subjected to centrifugation at 13,400 × *g* for 3 min. The supernatant was used as a template DNA in PCR assays.	Kuan et al., 2013
*Salmonella* spp., *S*. Enteritidis, and *S*. Typhimurium	Each MPN tube was centrifuged at 15,000 × *g* for 3 min. The supernatants were discarded and the pellet was resuspended in 200 μL Tris-EDTA buffer and boiled for 10 min. The mixture was then cooled at −20°C immediately for another 10 min, followed by centrifugation at 15,000 × *g* for 3 min. The supernatant was used as a template DNA in PCR assays.	Pui et al., 2011

**Table 2:** Target genes, primer sequences, PCR preparations, thermocycling conditions, and gel electrophoresis conditions used in this study.

**Target genes**	**Primer sequences**	PCR preparations^*^	**Thermocycling conditions**	**Gel electrophoresis conditions**	**Amplicon size**	**Reference(s)**
Shiga-toxin genes (*stx1* and *stx2*) were targeted for the detection of STEC	stx1-F: 5′-ATA AAT CGC CAT TCG TTG ACT AC-3′ stx1-R: 5′-AGA ACG CCC ACT GAG ATC ATC-3′ stx2-F: 5′-GGC ACT GTC TGA AAC TGC TCC-3′ stx2-R: 5′-TCG CCA GTT ATC TGA CAT TCT G-3′	5 μL 5 × PCR buffer, 2 μL 25 mM MgCl_2_, 0.5 μL 10 mM dNTP mix, 1 U Taq DNA Polymerase, 0.5 μM for each primer, 10.3 μL of sterile distilled water and 2 μL of extracted DNA.	Initialisation at 95°C for 5 min, 30 cycles of denaturation at 94°C for 30 s, annealing at 55°C for 30 s, elongation for 72°C for 45 s, and followed by final elongation at 72°C for 7 min.	An aliquot (5 μL) of the amplified PCR product was loaded and electrophoresed on 1.5% (w/v) agarose gel at 100 V for 28 min.	180 bp (*stx1*) and 255 bp (*stx2*)	ÇadIrc et al., 2010; Loo et al., 2013
*fliCh7* and *rfbO157* genes were targeted for the detection of O antigen and flagellar antigen in *E. coli* O157:H7	rfbO157-F: 5′-CGG ACA TCC ATG TGA TAT GG-3′ rfbO157-R: 5′-TTG CCT ATG TAC AGC TAA TCC-3′ fliCh7-F: 5′-GCG CTG TCG AGT TCT ATC GAG-3′ fliCh7-R: 5′-CAA CGG TGA CTT TAT CGC CAT TCC-3′	5 μL 5 × PCR buffer, 2 μL 25 mM MgCl_2_, 0.5 μL 10 mM dNTP mix, 1 U Taq DNA Polymerase, 0.5 μM for each primer, 10.3 μL of sterile distilled water and 2 μL of extracted DNA.	Initialisation at 94°C for 5 min, 35 cycles of denaturation at 94°C for 1 min, annealing at 55°C for 30 s, and elongation at 72°C for 1 min and followed by a final elongation at 72°C for 10 min.	An aliquot (5 μL) of the amplified PCR product was loaded and electrophoresed on 1.5% (w/v) agarose gel at 100 V for 28 min.	259 bp (*rfbO157*) and 625 bp (*fliCh7*)	Hashemi et al., 2010; Loo et al., 2013
16S rRNA gene was targeted for the detection of *Listeria* spp.*hlyA* gene was targeted for the detection of *L. monocytogenes*	U1: 5′-CTC CAT AAA GGT GAC CCT-3′ LI1: 5′-CAG CMG CCG CGG TAA TWC-3′ LM1: 5′-CCT AAG ACG CCA ATC GAA-3′ LM2: 5′-AAG CGC TTG CAA CTG CTC-3′	5 μL 5 × PCR buffer, 1.5 μL 25 mM MgCl_2_, 0.2 μL 10 mM deoxynucleoside triphosphate (dNTP) mix, 1.5 U Taq DNA Polymerase, 0.5 μM for each primer LM1 and LM2, 1.0 μM of each primer U1 and LI1, 14 μL of sterile distilled water and 2 μL of extracted DNA.	Initialisation at 94°C for 5 min, 30 cycles of denaturation at 94°C for 30 s, annealing at 53°C for 1 min, and elongation at 72°C for 2 min, and followed by a final elongation at 72°C for 7 min.	An aliquot (5 μL) of the amplified PCR product was loaded and electrophoresed on 1.0% (w/v) agarose gel at 100 V for 28 min.	938 bp 702 bp	Border et al., 1990; Kuan et al., 2013
Random fragment was targeted for the detection of *Salmonella* spp.	ST11: 5′-GCC AAC CAT TGC TAA ATT GGC GCA-3′ ST15: 5′-GGT AGA AAT TCC CAG CGG GTA CTG G-3′	5 μL 5 × PCR buffer, 2.5 μL 25 mM MgCl_2_, 0.5 μL 10 mM dNTP mix, 1.5 U Taq DNA polymerase, 0.5 μL primer mix, (0.2 μM for ST11 and ST15, 1.2 μM for Fli15, Typ04, ENTF, and ENTR) 14.2 μL sterile distilled water, and 2 μL of extracted DNA.	Initialisation at 94°C for 2 min, 30 cycles of denaturation at 94°C for 45 s, annealing at 53°C for 1 min, elongation at 72°C for 1 min, and followed by a final elongation at 72°C for 7 min.	An aliquot (5 μL) of the amplified PCR product was loaded and electrophoresed on 1.5% (w/v) agarose gel at 100 V for 40 min.	429 bp	Soumet et al., 1999; Alvarez et al., 2004; Pui et al., 2011; Thung et al., 2016
*SdfI* gene was targeted for the detection of *S*. Enteritidis	ENTF: 5′-TGT GTT TTA TCT GAT GCA AGA GG-3′ ENTR: 5′-TGA ACT ACG TTC GTT CTT CTG G-3′				304 bp	
*fliC* gene was targeted for the detection of *S*. Typhimurium	Fli15: 5′-CGG TGT TGC CCA GGT TGG TAA T-3′ Typ04: 5′-ACT GGT AAA GAT GGC T-3′				620 bp	

* *Amplification of DNA was performed in 25 μL reaction mixtures*.

### Statistical analysis

Colony counts were converted into log_10_ CFU/g. The data were subjected to a one-way analysis of variance (ANOVA) analysis using Minitab 16.0 software (Minitab Inc., State College, Pennsylvania, U.S.A.) to evaluate if there were differences between the organic and conventional vegetables at *P* ≤ 0.05 level of significance.

## Results

### Microbiological quality of conventional and organic vegetables

Table 3 shows the microbial counts of mesophilic aerobic bacteria, yeasts and molds, and total coliforms in 12 types of organic and conventional vegetables. Among the 12 types of vegetables analyzed, no trend was shown that either organic or conventional vegetable has a greater microbial count of mesophilic aerobic bacteria, yeasts and molds, and total coliforms. However, cabbage, carrot, and winged bean showed significant differences (*P* < 0.05) in mesophilic aerobic population between organic and conventional samples. For mesophilic aerobic bacteria, the results varied from 3 to >7 log_10_ CFU/g for organic vegetables and 3 to > 8 log_10_ CFU/g for conventional vegetables. Most of the samples had a mesophilic aerobic bacteria count that ranged from 5 to 7 log_10_ CFU/g.

**Table 3:** Microbial counts (log_10_ CFU/g) of mesophilic aerobic bacteria, yeasts and molds, and total coliforms in different organic and conventional vegetables purchased at retail markets in Malaysia.

**Samples**	**Mesophilic aerobic bacteria**		**Total coliforms**		**Yeasts and molds**	
	**Conventional**	**Organic**	**Conventional**	**Organic**	**Conventional**	**Organic**
Cabbage	6.97 ± 0.51	5.82 ± 0.71^*^	4.83 ± 2.89	2.28 ± 2.57	6.36 ± 1.07	5.40 ± 0.74
Calamondin	5.10 ± 0.61	4.56 ± 1.62	ND^a^	ND^a^	4.86 ± 0.79	4.84 ± 1.43
Carrot	5.57 ± 0.35	6.29 ± 0.41^*^	4.22 ± 0.89	5.62 ± 0.45^*^	5.59 ± 0.70	6.03 ± 0.51
Cherry tomato	3.90 ± 2.17	3.17 ± 2.46	2.09 ± 2.73	1.89 ± 2.10	1.51 ± 1.89	3.15 ± 2.50
Chili	8.06 ± 0.38	7.91 ± 0.55	7.04 ± 0.85	6.94 ± 0.61	5.95 ± 0.55	6.48 ± 1.18
Cucumber	5.55 ± 0.60	6.24 ± 0.53	2.48 ± 2.36	2.67 ± 3.07	3.35 ± 1.58	4.58 ± 0.91
Eggplant	6.89 ± 0.74	6.23 ± 0.49	5.18 ± 2.33	4.13 ± 2.09	5.53 ± 0.81	4.78 ± 0.76
Winged bean	7.41 ± 0.35	6.91 ± 0.17^*^	4.73 ± 2.22	3.49 ± 1.75	6.05 ± 0.30	5.61 ± 0.08^*^
Lettuce	6.70 ± 0.58	7.14 ± 0.60	4.61 ± 0.88	4.06 ± 2.14	5.77 ± 0.66	6.30 ± 0.70
Sweet potato	7.09 ± 0.28	6.50 ± 1.42	5.70 ± 0.69	4.05 ± 2.34	5.48 ± 0.35	4.84 ± 0.92
Tomato	4.90 ± 1.50	3.63 ± 3.02	1.00 ± 1.71	1.70 ± 2.77	0.48 ± 1.26	1.37 ± 2.14
White radish	7.76 ± 0.77	7.13 ± 0.39	6.11 ± 1.01	5.01 ± 1.35	6.90 ± 0.76	6.28 ± 0.60

*Mean comparisons between conventional and organic within mesophilic aerobic bacteria, total coliforms and yeasts and molds by one-way ANOVA at P ≤ 0.05. The results were expressed as mean ± SD (log_10_ CFU/g) of three measurements*.

* *Significantly different at P ≤ 0.05*.

a *ND: Not detectable in 25 g*.

For total coliforms counts, results varied from 1 to 7 log_10_ CFU/g for organic and conventional vegetables. In this study, no coliform bacterium was detected in calamondin samples and carrot was the only sample that showed significant differences (*P* < 0.05) in coliform populations between organic and conventional samples. Overall, greater microbial counts (mesophilic aerobic bacteria, yeasts and molds, and total coliforms) were detected in chili samples whereas lower microbial counts were found in cherry tomato and tomato samples.

The yeasts and molds counts in the vegetables were lower compared to mesophilic aerobic bacteria, ranged from 1 to > 6 log_10_ CFU/g for organic vegetables and 0.5 to > 6 log_10_ CFU/g for conventional vegetables. The yeasts and molds counts of most samples varied from 3 to 6 log_10_ CFU/g. In this study, all conventionally and organically grown vegetable samples showed comparable yeast and mold counts except for conventional winged bean which showed a higher count compared to the organic counterpart.

### Microbiological safety of conventional and organic vegetables

As shown in Tables 4–6, there was obviously not much difference in the prevalence of foodborne pathogens between conventional and organic vegetables. Therefore, statistical analysis for comparison of the positive-negative data for foodborne pathogens between conventional and organic vegetables was not conducted in this study. In this study, *E. coli* O157:H7 and *S*. Typhimurium were not detected in 152 vegetable samples (Tables 4, 5). Of the 152 samples analyzed, only the conventional lettuce (Table 4) and organic carrot (Table 5) were contaminated with STEC and *S*. Enteritidis, respectively. *L. monocytogenes* were more frequently detected in both organic and conventional vegetables as compared to *E. coli* O157:H7, *S*. Typhimurium, and *S*. Enteritidis (Tables 4–6). The prevalence of *L. monocytogenes* in conventional and organic vegetables was 9.1% (seven positive samples out of 77 samples) and 2.7% (two positive samples out of 75 samples), respectively. The microbial load of *L. monocytogenes* in vegetable samples ranged between < 3 and 6.0 MPN/g (Table 7). Overall, the microbial load for most positive samples was ranged from < 3 to 3.0 MPN/g.

**Table 4:** Prevalence of STEC O157:H7 and STEC non-O157 in conventional and organic vegetables purchased at retail markets in Malaysia using the MPN-PCR method.

**Types of samples**	**STEC O157:H7**						**STEC non-O157**					
	**Conventional**		**Organic**		**Total**		**Conventional**		**Organic**		**Total**	
	**(n_*p*_/n_*t*_)**	**%**	**(n_*p*_/n_*t*_)**	**%**	**(n_*p*_/n_*t*_)**	**%**	**(n_*p*_/n_*t*_)**	**%**	**(n_*p*_/n_*t*_)**	**%**	**(n_*p*_/n_*t*_)**	**%**
Cabbage	(0/5)	0	(0/6)	0	(0/11)	0	(0/5)	0	(0/6)	0	(0/11)	0
Calamondin	(0/6)	0	(0/7)	0	(0/13)	0	(0/6)	0	(0/7)	0	(0/13)	0
Carrot	(0/6)	0	(0/7)	0	(0/13)	0	(0/6)	0	(0/7)	0	(0/13)	0
Cherry tomato	(0/7)	0	(0/6)	0	(0/13)	0	(0/7)	0	(0/6)	0	(0/13)	0
Chili	(0/7)	0	(0/6)	0	(0/13)	0	(0/7)	0	(0/6)	0	(0/13)	0
Cucumber	(0/7)	0	(0/6)	0	(0/13)	0	(0/7)	0	(0/6)	0	(0/13)	0
Eggplant	(0/7)	0	(0/6)	0	(0/13)	0	(0/7)	0	(0/6)	0	(0/13)	0
Winged bean	(0/7)	0	(0/6)	0	(0/13)	0	(0/7)	0	(0/6)	0	(0/13)	0
Lettuce	(0/6)	0	(0/7)	0	(0/13)	0	(1/6)	16.7	(0/7)	0	(1/13)	7.7
Sweet potato	(0/6)	0	(0/6)	0	(0/12)	0	(0/6)	0	(0/6)	0	(0/12)	0
Tomato	(0/7)	0	(0/6)	0	(0/13)	0	(0/7)	0	(0/6)	0	(0/13)	0
White radish	(0/6)	0	(0/6)	0	(0/12)	0	(0/6)	0	(0/6)	0	(0/12)	0
Total	(0/77)	0	(0/75)	0	(0/152)	0	(1/77)	1.3	(0/75)	0	(1/152)	0.7

*n_p_, Total number of positive samples confirmed by MPN-PCR; n_t_, Total number of samples; %, Percentage of positive sample*.

**Table 5:** Prevalence of *Salmonella* spp., *S*. Enteritidis, and *S*. Typhimurium in conventional and organic vegetables purchased at retail markets in Malaysia using the MPN-PCR method.

**Types of samples**	***Salmonella*** **spp**.						***S***. **Enteritidis**						***S***. **Typhimurium**					
	**Conventional**		**Organic**		**Total**		**Conventional**		**Organic**		**Total**		**Conventional**		**Organic**		**Total**	
	**(n_*p*_/n_*t*_)**	**%**	**(n_*p*_/n_*t*_)**	**%**	**(n_*p*_/n_*t*_)**	**%**	**(n_*p*_/n_*t*_)**	**%**	**(n_*p*_/n_*t*_)**	**%**	**(n_*p*_/n_*t*_)**	**%**	**(n_*p*_/n_*t*_)**	**%**	**(n_*p*_/n_*t*_)**	**%**	**(n_*p*_/n_*t*_)**	**%**
Cabbage	(0/5)	0	(0/6)	0	(0/11)	0	(0/5)	0	(0/6)	0	(0/11)	0	(0/5)	0	(0/6)	0	(0/11)	0
Calamondin	(0/6)	0	(1/7)	14.3	(1/13)	7.7	(0/6)	0	(0/7)	0	(0/13)	0	(0/6)	0	(0/7)	0	(0/13)	0
Carrot	(0/6)	0	(1/7)	14.3	(1/13)	7.7	(0/6)	0	(1/7)	14.3	(1/13)	7.7	(0/6)	0	(0/7)	0	(0/13)	0
Cherry tomato	(0/7)	0	(0/6)	0	(0/13)	0	(0/7)	0	(0/6)	0	(0/13)	0	(0/7)	0	(0/6)	0	(0/13)	0
Chili	(0/7)	0	(0/6)	0	(0/13)	0	(0/7)	0	(0/6)	0	(0/13)	0	(0/7)	0	(0/6)	0	(0/13)	0
Cucumber	(0/7)	0	(1/6)	16.7	(1/13)	7.7	(0/7)	0	(0/6)	0	(0/13)	0	(0/7)	0	(0/6)	0	(0/13)	0
Eggplant	(0/7)	0	(0/6)	0	(0/13)	0	(0/7)	0	(0/6)	0	(0/13)	0	(0/7)	0	(0/6)	0	(0/13)	0
Winged bean	(0/7)	0	(0/6)	0	(0/13)	0	(0/7)	0	(0/6)	0	(0/13)	0	(0/7)	0	(0/6)	0	(0/13)	0
Lettuce	(0/6)	0	(0/7)	0	(0/13)	0	(0/6)	0	(0/7)	0	(0/13)	0	(0/6)	0	(0/7)	0	(0/13)	0
Sweet potato	(0/6)	0	(0/6)	0	(0/12)	0	(0/6)	0	(0/6)	0	(0/12)	0	(0/6)	0	(0/6)	0	(0/12)	0
Tomato	(0/7)	0	(0/6)	0	(0/13)	0	(0/7)	0	(0/6)	0	(0/13)	0	(0/7)	0	(0/6)	0	(0/13)	0
White radish	(0/6)	0	(0/6)	0	(0/12)	0	(0/6)	0	(0/6)	0	(0/12)	0	(0/6)	0	(0/6)	0	(0/12)	0
Total	(0/77)	0	(3/75)	4.0	(3/152)	2.0	(0/77)	0	(1/75)	1.3	(1/152)	0.7	(0/77)	0	(0/75)	0	(0/152)	0

*n_p_, Total number of positive samples confirmed by MPN-PCR; n,Total number of samples; %, Percentage of positive sample*.

**Table 6:** Prevalence of *Listeria* spp. and *L. monocytogenes* in conventional and organic vegetables purchased at retail markets in Malaysia using the MPN-PCR method.

**Types of samples**	***Listeria*** **spp**.						***L. monocytogenes***					
	**Conventional**		**Organic**		**Total**		**Conventional**		**Organic**		**Total**	
	**(n_*p*_/n_*t*_)**	**%**	**(n_*p*_/n_*t*_)**	**%**	**(n_*p*_/n_*t*_)**	**%**	**(n_*p*_/n_*t*_)**	**%**	**(n_*p*_/n_*t*_)**	**%**	**(n_*p*_/n_*t*_)**	**%**
Cabbage	(1/5)	20.0	(1/6)	16.7	(2/11)	18.2	(1/5)	20.0	(1/6)	16.7	(2/11)	18.2
Calamondin	(1/6)	16.7	(0/7)	0	(1/13)	7.7	(1/6)	16.7	(0/7)	0	(1/13)	7.7
Carrot	(1/6)	16.7	(0/7)	0	(1/13)	7.7	(1/6)	16.7	(0/7)	0	(1/13)	7.7
Cherry tomato	(0/7)	0	(0/6)	0	(0/13)	0	(0/7)	0	(0/6)	0	(0/13)	0
Chili	(3/7)	42.9	(0/6)	0	(3/13)	23.1	(1/7)	14.3	(0/6)	0	(1/13)	7.7
Cucumber	(1/7)	14.3	(0/6)	0	(1/13)	7.7	(1/7)	14.3	(0/6)	0	(1/13)	7.7
Eggplant	(0/7)	0	(0/6)	0	(0/13)	0	(0/7)	0	(0/6)	0	(0/13)	0
Winged bean	(1/7)	14.3	(0/6)	0	(1/13)	7.7	(1/7)	14.3	(0/6)	0	(1/13)	7.7
Lettuce	(1/6)	16.7	(2/7)	28.6	(3/13)	23.1	(1/6)	16.7	(0/7)	0	(1/13)	7.7
Sweet potato	(0/6)	0	(0/6)	0	(0/12)	0	(0/6)	0	(0/6)	0	(0/12)	0
Tomato	(0/7)	0	(0/6)	0	(0/13)	0	(0/7)	0	(0/6)	0	(0/13)	0
White radish	(0/6)	0	(2/6)	33.3	(2/12)	16.7	(0/6)	0	(1/6)	16.7	(1/12)	8.3
Total	(9/77)	11.7	(5/75)	6.7	(14/152)	9.2	(7/77)	9.1	(2/75)	2.7	(9/152)	5.9

*n_p_, Total number of positive samples confirmed by MPN-PCR; n_t_, Total number of samples; %, Percentage of positive sample*.

**Table 7:** Prevalence and microbial load (MPN/g) of STEC non-O157:H7, *L. monocytogenes* and *S*. Enteritidis in organic and conventional vegetables purchased at retail markets in Malaysia.

**Types of vegetables**	**STEC non-O157:H7**				***L. monocytogenes***				***S***. **Enteritidis**			
	**Conventional**		**Organic**		**Conventional**		**Organic**		**Conventional**		**Organic**	
	**Prevalence (%)**	**Microbial load (MPN/g)**	**Prevalence (%)**	**Microbial load (MPN/g)**	**Prevalence (%)**	**Microbial load (MPN/g)**	**Prevalence (%)**	**Microbial load (MPN/g)**	**Prevalence (%)**	**Microbial load (MPN/g)**	**Prevalence (%)**	**Microbial load (MPN/g)**
Cabbage	0	< 3.0	0	< 3.0	20.0	< 3.0–3.0	16.7	< 3.0–3.0	0	< 3.0	0	< 3.0
Calamondin	0	< 3.0	0	< 3.0	16.7	< 3.0–6.0	0	< 3.0	0	< 3.0	0	< 3.0
Carrot	0	< 3.0	0	< 3.0	16.7	< 3.0–3.0	0	< 3.0	0	< 3.0	14.3	< 3.0–3.0
Cherry tomato	0	< 3.0	0	< 3.0	0	< 3.0	0	< 3.0	0	< 3.0	0	< 3.0
Chili	0	< 3.0	0	< 3.0	14.3	< 3.0–3.0	0	< 3.0	0	< 3.0	0	< 3.0
Cucumber	0	< 3.0	0	< 3.0	14.3	< 3.0-3.0	0	< 3.0	0	< 3.0	0	< 3.0
Eggplant	0	< 3.0	0	< 3.0	0	< 3.0	0	< 3.0	0	< 3.0	0	< 3.0
Winged bean	0	< 3.0	0	< 3.0	14.3	< 3.0–3.0	0	< 3.0	0	< 3.0	0	< 3.0
Lettuce	16.7	< 3.0–> 1100	0	< 3.0	16.7	< 3.0–3.0	0	< 3.0	0	< 3.0	0	< 3.0
Sweet potato	0	< 3.0	0	< 3.0	0	< 3.0	0	< 3.0	0	< 3.0	0	< 3.0
Tomato	0	< 3.0	0	< 3.0	0	< 3.0	0	< 3.0	0	< 3.0	0	< 3.0
White radish	0	< 3.0	0	< 3.0	0	< 3.0	16.7	< 3.0–3.0	0	< 3.0	0	< 3.0
Total	1.3	< 3.0–> 1100	0	< 3.0	9.1	< 3.0–6.0	2.7	< 3.0–3.0	0	< 3.0	1.3	< 3.0–3.0

## Discussion

Since vegetables are grown in soil and exposed to different kind of environmental conditions and hazards, these conditions would be reflected in the mesophilic aerobic count. Therefore, the mesophilic aerobic count can be used as an indicator to access the microbiological quality of foods (Pianetti et al., 2008). Brackett and Splittstoesser (1992) found that mesophilic aerobic counts in vegetables can be as high as 7 log_10_ CFU/g. A previous study suggested that fruits and vegetables grown without or under low level of pesticides can be contaminated with larger microbial population since some pesticides have been found to inhibit the growth of some microorganisms (Guan et al., 2001). Oliveira et al. (2010) also reported that organic lettuce contained larger mesophilic aerobic population than conventional lettuce. Surprisingly, nine out of 12 types of organically and conventionally grown vegetables in this study showed comparable and no significant difference (*P* > 0.05) in mesophilic aerobic population.

Our findings were in tandem with the data obtained from previous studies (Oliveira et al., 2010; Maffei et al., 2013) in which yeasts and molds counts were lower than mesophilic aerobic bacteria counts. Yeasts and molds, depending on genus and species, are the main culprit in most fresh produce spoilage and can be pathogenic. These microbial groups can invade fresh produce in the field prior to harvest and during storage. The presence of yeasts and molds not only link to food spoilage problems in vegetable, they can also pose health risks due to mycotoxins production (Tournas, 2005; Tournas and Katsoudas, 2005). Diseases caused by exposure to mycotoxins include allergic reactions, immunosuppressive diseases, and possibly cancers (Kovács, 2004; Buyukunal et al., 2015).

Coliform bacteria are commonly used as an indicator of sanitary quality of foods or to check potential contamination of pathogenic microorganisms (Kornacki and Johnson, 2001). In this study, the presence of coliform bacteria in vegetable samples suggested the deterioration of the quality of vegetable due to fecal contamination. It may be caused by different contamination sources, such as the use of polluted irrigation water during pre-harvest, transportation, improper storage conditions, or poor handling practices along the entire food chain (National Advisory Committee on Microbiological Criteria for Foods, 1999). Despite most of the coliform bacteria do not cause disease, uncommon strain, such as *E. coli* O157:H7 is pathogenic to human which contributed toward many foodborne disease outbreaks (Delaquis et al., 2007; Cieslik and Bartoszcze, 2011; Chang et al., 2013). Interestingly, no coliform bacterium was found in both organic and conventional calamondin samples. This might due to the bioactive compounds on the peel of calamondin that protect it from microbial deterioration (Jeong et al., 2004; Rubiatul et al., 2015). Also, the acidic internal environment of calamondin does not favor the growth of coliform bacteria. Although most of the pathogens are distributed on the surface of fresh produce, contamination might occur through the internalization of opportunistic pathogens or spoilage bacteria into fresh produce. Montville and Matthews (2008) and Ryser et al. (2009) pointed out that microorganisms can gain access to the internal tissues of fresh produce via stomata, lenticels, trichomes, lesions caused by plant pathogens, and stem scars. Internalization of pathogens, such as *Salmonella* spp. and *E. coli* O157:H7 in fresh produce also have been widely reported (Bordini et al., 2007; Deering et al., 2012; Ge et al., 2012; Najwa et al., 2015; Nicholson et al., 2015).

In this study, emphasis was given to the detection of *S*. Typhimurium and *S*. Enteritidis among 2,463 serovars of *Salmonella* species. This was mainly due to *S*. Typhimurium and *S*. Enteritidis have been reported to be the most prevalent serovars and common causes of human of salmonellosis (Herikstad et al., 2002; Bangtrakulnonth et al., 2004; Rabsch et al., 2013; Najwa et al., 2015). Hence, it is our interest to investigate the occurrence of these two serovars in Malaysia. Also, based on the previous prevalence studies and epidemiological data, *S*. Typhimurium and *S*. Enteritidis are the common foodborne pathogens detected in Malaysia (Modarressi and Thong, 2010; Nillian et al., 2011; Pui et al., 2011; Adzitey et al., 2012; Najwa et al., 2015; Thung et al., 2016). It is worth noting that *E. coli* O157:H7 and *S*. Typhimurium were not detected in any samples. According to the study by Ryu et al. (2014), neither *E. coli* O157:H7 nor *Salmonella* spp. were detected in conventional and fresh organic produce. In another study conducted by Mukherjee et al. (2004), organic and conventional fresh produce in Minnesota, United States were found to be negative for *Salmonella* but positive for *E. coli* O157:H7. Although *E. coli* O157:H7 was not detected in all the samples, one of the conventional lettuce samples was contaminated with STEC. STEC are well-known as important pathogenic bacteria causing many foodborne illness outbreaks that are linked to consumption of raw vegetables (Loo et al., 2013). STEC can produce Shiga toxin which causes severe bloody diarrhea and results in life-threatening complications, such as haemolytic-uremic syndrome (HUS) (Mead and Griffin, 1998; Sarimehmetoglu et al., 2009).

The overall prevalence of foodborne pathogens in fresh produce (including conventional and fresh organic produce) were 0.7, 9.2, 5.9, 2.0, and 0.7% for STEC non-O157, *Listeria* spp., *L. monocytogenes, Salmonella* spp., and *S*. Enteritidis, respectively, which were comparatively lower as compare to previous local studies (Arumugaswamy et al., 1994; Jeyaletchumi et al., 2010; Chang et al., 2013; Loo et al., 2013; Najwa et al., 2015). These findings are also contrary to the findings by Oliveira et al. (2010) that no pathogen was found in 72 organically and conventionally grown lettuces. In this study, contaminations by *Listeria* spp. and *L. monocytogenes* in both conventional and organic vegetables were obviously observed, and being slightly higher in the conventional vegetables than in the organic vegetables. Although there was a low microbial load of *L. monocytogenes* in the fresh produce (ranging between < 3 and 6.0 MPN/g) and listeriosis cases are also rarely reported in Malaysia, it may pose a safety risk to consumers as a warm and humid environment may encourage proliferation of *L. monocytogenes* to a dangerous level in vegetables (Steinbruegge et al., 1988).

In this study, the comparison of microbiological quality and safety of organic and conventional vegetables showed no trend whether conventional fresh produce is more or less safe than organic ones. Regardless of the cultivation methods, fresh produce can be contaminated starting from the pre-harvest stage, for example, through the use of fresh or non-composted animal manure, irrigation water, wild animals, pests, and insects (Beuchat and Ryu, 1997; Mandrell, 2009; Talley et al., 2009; Mishra et al., 2017). Post-harvest handling activities, such as selection, trimming, precooling, washing, grading, packaging, storage, and transportation can exacerbate the situation (Mandrell, 2009; Buchholz et al., 2012; Maffei et al., 2013).

Additionally, the differences in the contamination levels of vegetables can be affected by farm location, weather or climatic conditions, and types of vegetable crops (e.g., leafy and salad, bulb and stem, root and tuber, flower and flower buds, seed and fruit) (Ryu et al., 2014). In Malaysia, the great difference in price between organic and conventional fresh produce by as much as 100–300%, indirectly may affect the microbiological quality of vegetables. Since fresh organic produce is sold at higher prices compared to those of conventional produce, farmers or retailers tend to use better post-harvest handling practices and higher quality packaging materials for organic produce. As a result, quality and safety of organic vegetables can be preserved.

## Conclusions

Findings in this study indicated that regardless of farming methods, either organic or conventional, raw vegetables can act as a potential vehicle for transmission of *Salmonella, L. monocytogenes*, and *E. coli* O157:H7 and thus, pose a health risk to consumers. Although the use of composted manure as a nutrient source for fresh produce in organic farming is believed to pose a greater risk of microbial contamination, this research found that one particular type of cultivation practices would not affect the microbiological status of fresh produce. However, more works are required to verify the observations in this study. Environmental factors, such as weather conditions and the post-harvest handling practices along the entire food chain should also be taken into account in future studies, since they may also affect the microbial level of organic and conventional fresh produce. The present study provides baseline information on the microbial profiles of organically and conventionally grown vegetables in Malaysia. Meanwhile, the data obtained in this study also serves as useful information in future risk assessment.

## Author contributions

CHK, YR, SA, CW, and SR developed the study design. CHK, CSK, and SY co-ordinated the collection of samples in retail markets required for this study. CHK, TT, JP, WC, YL, CT, OR, and SM conducted the microbiological analysis of food samples and carried out the PCR confirmation of specific foodborne pathogens, for example, *Listeria* spp. and *L. monocytogenes*, STEC, *E. coli* O157:H7, *Salmonella* spp., *S*. Enteritidis, and *S*. Typhimurium. MN provided culture media, PCR reagents, and technical advice on the study. CHK interpreted the data, drafted the manuscript, and revised the manuscript. YR, SA, CW, CSK, SY, and SR vetted the manuscript. All authors read and approved the final version of the manuscript.

### Conflict of interest statement

The authors declare that the research was conducted in the absence of any commercial or financial relationships that could be construed as a potential conflict of interest.

## References

[B1] Adzitey F. Rusul G. Huda N. (2012). Prevalence and antibiotic resistance of *Salmonella* serovars in ducks, duck rearing and processing environments in Penang, Malaysia. Food Res. Int. 45, 947–952. 10.1016/j.foodres.2011.02.051 22285201

[B2] Alvarez J. Sota M. Vivanco A. B. Perales I. Cisterna R. Rementeria A.. (2004). Development of a multiplex PCR technique for detection and epidemiological typing of *Salmonella* in human clinical samples. J. Clin. Microbiol. 42, 1734–1738. 10.1128/JCM.42.4.1734-1738.2004 15071035 PMC387595

[B3] Arumugaswamy R. K. Ali G. R. Abd Hamid S. N. (1994). Prevalence of *Listeria monocytogenes* in foods in Malaysia. Int. J. Food Microbiol. 23, 117–121. 10.1016/0168-1605(94)90227-5 7811569

[B4] Bangtrakulnonth A. Pornreongwong S. Pulsrikarn C. Sawanpanyalert P. Hendriksen R. S. Wong D. M.. (2004). *Salmonella* serovars from humans and other sources in Thailand, 1993–2002. Emerg. Infect. Dis. 10, 131–136. 10.3201/eid1001.02-0781 15078609 PMC3322742

[B5] Beuchat L. R. (2002). Ecological factors influencing survival and growth of human pathogens on raw fruits and vegetables. Microb. Infect. 4, 413–423. 10.1016/S1286-4579(02)01555-1 11932192

[B6] Beuchat L. R. Cousin M. A. (2001). Yeasts and molds, in Compendium of Methods for the Microbiological Examination of Foods, 4th Edn., eds Downes F. P. Ito K. (Washington, DC: American Public Health Association), 209–215.

[B7] Beuchat L. R. Ryu J. H. (1997). Produce handling and processing practices. Emerg. Infect. Dis. 3, 459–465. 10.3201/eid0304.970407 9366597 PMC2640071

[B8] Border P. M. Howard J. J. Plastow G. S. Siggens K. W. (1990). Detection of *Listeria* species and *Listeria monocytogenes* using polymerase chain reaction. Lett. Appl. Microbiol. 11, 158–162. 10.1111/j.1472-765X.1990.tb00149.x 1367467

[B9] Bordini M. E. B. Ristori C. A. Jakabi M. Gelli D. S. (2007). Incidence, internalization and behavior of *Salmonella* in mangoes, var. Tommy Atkins. Food Control. 18, 1002–1007. 10.1016/j.foodcont.2006.06.003

[B10] Brackett R. E. Splittstoesser D. F. (1992). Fruits and vegetables, in Compendium of Methods for the Microbiological Examination of Food, 3rd Edn., eds Vanderzant C. Splittstoesser D. F. (Washington, DC: American Public Health Association), 919–927.

[B11] Buchholz A. L. Davidson G. R. Marks B. P. Todd E. C. Ryser E. T. (2012). Quantitative transfer of *Escherichia coli* O157:H7 to equipment during small scale production of fresh-cut leafy greens. J. Food Prot. 75, 1184–1197. 10.4315/0362-028X.JFP-11-489 22980000

[B12] Buyukunal S. K. Issa G. Aksu F. Vural A. (2015). Microbiological quality of fresh vegetables and fruits collected from supermarkets in Istanbul, Turkey. J. Food Nutr. Sci. 3, 152–159. 10.11648/j.jfns.20150304.13

[B13] ÇadIrc I. O. SirIken B. Inat G. Kevenk T. O. (2010). The prevalence of *Escherichia coli* O157 and O157:H7 in ground beef and raw meatball by immunomagnetic separation and the detection of virulence genes using multiplex PCR. Meat Sci. 84, 553–556. 10.1016/j.meatsci.2009.10.011 20374823

[B14] Centers for Disease Control and Prevention (2011). Multistate Outbreak of Human Salmonella Enteritidis Infections Linked to Alfalfa Sprouts and Spicy Sprouts (Final Update). Available Online at: http://www.cdc.gov/salmonella/2011/alfalfa-spicy-sprouts-7-6-2011.html (Accessed December 11, 2016).

[B15] Centers for Disease Control and Prevention (2012). Multistate Outbreak of E. coli O157:H7 Infections Linked to Romaine Lettuce (Final Update). Available Online at: http://www.cdc.gov/ecoli/2011/romaine-lettace-3-23-12.html (Accessed December 11, 2016).

[B16] Chang W. S. Afsah-Hejri L. Rukayadi Y. Khatib A. Lye Y. L. Loo Y. Y. (2013). Quantification of *Escherichia coli* O157:H7 in organic vegetables and chickens. Int. Food Res. J. 20, 1023–1029.

[B17] Cieslik P. Bartoszcze M. (2011). Enterohaemorrhagic *Escherichia coli* (EHEC) infections: a threat to public health. Med. Weter 67, 571–578.

[B18] Deering A. J. Mauer L. J. Pruitt R. E. (2012). Internalization of *E. coli* O157: H7 and *Salmonella* spp. in plants: a review. Food Res. Int. 45, 567–575. 10.1016/j.foodres.2011.06.058

[B19] Delaquis P. Bach S. Dinu L. D. (2007). Behavior of *Escherichia coli* O157:H7 in leafy vegetables. J. Food Prot. 70, 1966–1974. 10.4315/0362-028X-70.8.1966 17803159

[B20] Ge C. Lee C. Lee J. (2012). The impact of extreme weather events on *Salmonella* internalization in lettuce and green onion. Food Res. Int. 45, 1118–1122. 10.1016/j.foodres.2011.06.054

[B21] Guan T. Y. Blank G. Ismond A. Van Acker R. (2001). Fate of foodborne bacterial pathogens in pesticide products. J. Sci. Food Agr. 81, 503–512. 10.1002/jsfa.835

[B22] Hashemi M. Khanzadi S. Jamshidi A. (2010). Identification of *Escherichia coli* O157:H7 isolated from Cattle Carcasses in Mashhad Abattoir by multiplex PCR. World Appl. Sci. J. 10, 703–708.

[B23] Herikstad H. Motarjemi Y. Tauxe R. V. (2002). *Salmonella* surveillance: a global survey of public health serotyping. Epidemiol. Infect. 129, 1–8. 10.1017/S0950268802006842 12211575 PMC2869853

[B24] Institute of Food Technologists (2000). IFT Expert Report on Emerging Microbiological Food Safety Issues-Implications for Control in the 21st Century. Available Online at: http://www.ift.org/~/media/Knowledge%20Center/Science%20Reports/Expert%20Reports/Emerging%20Microbiological/Emerging%20Micro.pdf (Accessed April 27 2017).

[B25] Jeong S. M. Kim S. Y. Kim D. R. Jo S. C. Nam K. C. Ahn D. U.. (2004). Effect of heat treatment on the antioxidant activity of extracts from citrus peels. J. Agric. Food Chem. 52, 3389–3393. 10.1021/jf049899k 15161203

[B26] Jeyaletchumi P. Tunung R. Margaret S. P. Son R. Ghazali F. M. Cheah Y. K. (2010). *Listeria monocytogenes* in raw salad vegetables sold at retail level in malaysia. Food Control 21, 774–778. 10.1016/j.foodcont.2009.09.008

[B27] Johannessen G. S. Froseth R. B. Solemdal L. Jarp J. Wasteson Y. Rorvik L. M. (2004). Influence of bovine manure as fertilizer on the bacteriological quality of organic Iceberg lettuce. J. Appl. Microbiol. 96, 787–794. 10.1111/j.1365-2672.2004.02208.x 15012817

[B28] Kornacki J. L. Johnson J. L. (2001). Enterobacteriaceae, coliforms, and Escherichia coli as quality and safety indicators, in Compendium of Methods for the Microbiological Examination of Foods, 4th Edn., eds Downes F. P. Ito K. (Washington, DC: American Public Health Association), 69–82.

[B29] Kovács M. (2004). Nutritional health aspects of mycotoxins. Orv. Hetil. 145, 1739–1746. 15493122

[B30] Kuan C. H. Goh S. G. Loo Y. Y. Chang W. S. Lye Y. L. Soopna P.. (2013). Prevalence and quantification of *Listeria monocytogenes* in chicken offal at the retail level in Malaysia. Poult. Sci. 92, 1664–1669. 10.3382/ps.2012-02974 23687164

[B31] Loo Y. Y. Puspanadan S. Goh S. G. Kuan C. H. Chang W. S. Lye Y. L. (2013). Quantitative detection and characterization of Shiga toxin-producing *Escherichia coli* O157 and non-O157 in raw vegetables by MPN-PCR in Malaysia. Int. Food Res. J. 20, 3313–3317.

[B32] Maffei D. F. Silveira N. F. A. Catanozi M. P. L. M. (2013). Microbiological quality of organic and conventional vegetables sold in Brazil. Food Control. 29, 226–230. 10.1016/j.foodcont.2012.06.013

[B33] Magkos F. Arvaniti F. Zampelas A. (2006). Organic food: buying more safety or just peace of mind? a critical review of the literature. Crit. Rev. Food Sci. Nutr. 46, 23–56. 10.1080/10408690490911846 16403682

[B34] Mandrell R. E. (2009). Enteric human pathogens associated with fresh produce: sources, transport, and ecology, in Microbial Safety of Fresh Produce, eds Fan X. Niemira B. Doona C. J. Feeherry F. Gravani R. B. (Ames, IA: IFT Press/Wiley-Blackwell Publishing), 5–41.

[B35] McMahon M. A. S. Wilson I. G. (2001). The occurrence of enteric pathogens and *Aeromonas* species in organic vegetables. Int. J. Food Microbiol. 70, 155–162. 10.1016/S0168-1605(01)00535-9 11759753

[B36] Mead P. S. Griffin P. M. (1998). *Escherichia coli* O157:H7. Lancet 352, 1207–1212. 10.1016/S0140-6736(98)01267-7 9777854

[B37] Mishra A. Guo M. Buchanan R. L. Schaffner D. W. Pradhan A. K. (2017). Development of growth and survival models for *Salmonella* and *Listeria monocytogenes* during non-isothermal time-temperature profiles in leafy greens. Food Control 71, 32–41. 10.1016/j.foodcont.2016.06.009

[B38] Modarressi S. Thong K. L. (2010). Isolation and molecular sub typing of *Salmonella enterica* from chicken, beef and street foods in Malaysia. Sci. Res. Essays 5, 2713–2720.

[B39] Mohamad S. S. Rusdi S. D. Hashim N. H. (2014). Organic food consumption among urban consumers: preliminary results. Procedia. Soc. Behav. Sci. 130, 509–514. 10.1016/j.sbspro.2014.04.059

[B40] Montville T. J. Matthews K. R. (2008). Enterohemorrhagic Escherichia coli, in Food Microbiology: an Introduction, 2nd Edn., eds Montville T. J. Matthews K. R. (Washington, DC: ASM Press), 123–140.

[B41] Morton R. D. (2001). Aerobic plate count, in Compendium of Methods for the Microbiological Examination of Foods, 4th Edn., eds Downes F. P. Ito K. (Washington, DC: American Public Health Association), 63–67.

[B42] Mukherjee A. Speh D. Dyck E. Diez-Gonzalez F. (2004). Preharvest evaluation of coliforms, *Escherichia coli, Salmonella*, and *Escherichia coli* O157:H7 in organic and conventional produce grown by Minnesota farmers. J. Food Prot. 67, 894–900. 10.4315/0362-028X-67.5.894 15151224

[B43] Najwa M. S. Rukayadi Y. Ubong A. Loo Y. Y. Chang W. S. Lye Y. L. (2015). Quantification and antibiotic susceptibility of *Salmonella* spp., *Salmonella* Enteritidis and *Salmonella* Typhimurium in raw vegetables(ulam). Int. Food Res. J. 22, 1761–1769.

[B44] National Advisory Committee on Microbiological Criteria for Foods (1999). Microbiological safety evaluations and recommendations on fresh produce. Food Control 10, 117–143. 10.1016/S0956-7135(99)00026-2

[B45] Nicholson A. M. Gurtler J. B. Bailey R. B. Niemira B. A. Douds D. D. (2015). Influence of mycorrhizal fungi on fate of *E. coli* O157: H7 and *Salmonella* in soil and internalization into Romaine lettuce plants. Int. J. Food Microbiol. 192, 95–102. 10.1016/j.ijfoodmicro.2014.10.001 25440552

[B46] Nillian E. Chai L. C. Pui C. F. Tunung R. Ubong A. Tuan Zainazor T. C. (2011). Simultaneous detection of *Salmonella* spp., *Salmonella* Enteritidis and *Salmonella* Typhimurium in raw salad vegetables and vegetarian burger patties. Food Nutr. Sci. 2, 1077–1081. 10.4236/fns.2011.210144

[B47] Olaimat A. N. Holley R. A. (2012). Factors influencing the microbial safety of fresh produce: a review. Food Microbiol. 32, 1–19. 10.1016/j.fm.2012.04.016 22850369

[B48] Oliveira M. Usall J. Vinas I. Anguera M. Gatius F. Abadias M. (2010). Microbiological quality of fresh lettuce from organic and conventional production. Food Microbiol. 27, 679–684. 10.1016/j.fm.2010.03.008 20510788

[B49] Pell A. N. (1997). Manure and microbes: public and animal health problem? J. Dairy Sci. 80, 2673–2681. 10.3168/jds.S0022-0302(97)76227-1 9361239 PMC7130904

[B50] Pianetti A. Sabatini L. Citterio B. Pierfelici L. Ninfali P. Bruscolini F. (2008). Changes in microbial populations in ready-to-eat vegetable salads during shelf-life. Ital. J. Food Sci. 20, 245–254.

[B51] Pui C. F. Wong W. C. Chai L. C. Nillian E. Mohamad Ghazali F. Cheah Y. K. (2011). Simultaneous detection of *Salmonella* spp., *Salmonella* Typhi and *Salmonella* Typhimurium in sliced fruits using multiplex PCR. Food Control 22, 337–342. 10.1016/j.foodcont.2010.05.021

[B52] Rabsch W. Simon S. Humphrey T. (2013). Public health aspects of *Salmonella* infections, in Salmonella in Domestic Animals, 2nd Edn., ed Barrow P. A. Methner U. (Wallingsford: CABI), 351–376.

[B53] Rubiatul A. S. Nor Helya I. K. Zarina Z. Dachyar A. Nurul Ain H. A. (2015). Antibacterial properties of limau kasturi (*C. microcarpa*) peels extract. Adv. Environ. Biol. 9, 5–9.

[B54] Ryser E. T. Jao J. Yan Z. (2009). Internalization of Pathogens in Produce in Microbial Safety of Fresh Produce, ed Fan X. Brendan B. A. Doona C. J. Feeherry F. E. Gravani R. B. (Ames, IA: John Wiley & Sons Inc.), 55–80.

[B55] Ryu J. H. Kim M. Kim E. G. Beuchat L. R. Kim H. (2014). Comparison of the microbiological quality of environmentally friendly and conventionally grown vegetables sold at retail markets in Korea. J. Food Sci. 79, 1739–1744. 10.1111/1750-3841.12531 25124136

[B56] Sarimehmetoglu B. Aksoy M. H. Ayaz N. D. Ayaz Y. Kuplulu O. Kaplan Y. Z. (2009). Detection of *Escherichia coli* O157:H7 in ground beef using immunomagnetic separation and multiplex PCR. Food Control 20, 357–361. 10.1016/j.foodcont.2008.06.002

[B57] Somasundram C. Razali Z. Santhirasegaram V. (2016). A Review on organic food production in Malaysia. Hort 2, 1–5. 10.3390/horticulturae2030012

[B58] Soumet C. Ermel G. Rose N. Rose V. Drouin P. Salvat G.. (1999). Evaluation of a multiplex PCR assay for simultaneous identification of *Salmonella* sp., *Salmonella* Enteritidis and *Salmonella* Typhimurium from environmental swabs of poultry houses. Lett. Appl. Microbiol. 28, 113–117. 10.1046/j.1365-2672.1999.00488.x 10063640

[B59] Steinbruegge E. G. Maxcy R. B. Liewen M. B. (1988). Fate of *Listeria monocytogenes* on ready-to-serve lettuce. J. Food Prot. 51, 596–599. 10.4315/0362-028X-51.8.596 30991601

[B60] Stephenson J. (1997). Public health experts take aim at a moving target: foodborne infections. J. Am. Med. Assoc. 277, 97–98. 10.1001/jama.1997.03540260011004 8990315

[B61] Talley J. L. Wayadande A. C. Wasala L. P. Gerry A. C. Fletcher J. DeSilva U.. (2009). Association of *Escherichia coli* O157:H7 with filth flies (*Muscidae* and *Calliphoridae*) captured in leafy greens fields and experimental transmission of *E. coli* O157:H7 to spinach leaves by house flies. J. Food Prot. 72, 1547–1552. 10.4315/0362-028X-72.7.1547 19681284

[B62] Thung T. Y. Mahyudin N. A. Basri D. F. Wan Mohamed Radzi C. W. J. Nakaguchi Y. Nishibuchi M.. (2016). Prevalence and antibiotic resistance of *Salmonella* Enteritidis and *Salmonella* Typhimurium in raw chicken meat at retail markets in Malaysia. Poult. Sci. 95, 1888–1893. 10.3382/ps/pew144 27118863

[B63] Tiraieyari N. Hamzah A. Samah B. A. (2014). Organic farming and sustainable agriculture in Malaysia: organic farmers' challenges towards adoption. Asian Soc. Sci. 10, 1–7. 10.5539/ass.v10n4p1

[B64] Tournas V. H. (2005). Molds and yeasts in fresh and minimally processed vegetables, fruits, and sprouts. Int. J. Food Microbiol. 99, 71–77. 10.1016/j.ijfoodmicro.2004.08.009 15718030

[B65] Tournas V. H. Katsoudas E. (2005). Mould and yeast flora in fresh berries, grapes and citrus fruits. Int. J. Food Microbiol. 105, 11–17. 10.1016/j.ijfoodmicro.2005.05.002 16023239

[B66] Willer H. Kilcher L. (2009). The World of Organic Agriculture-Statistics and Emerging Trends 2009. FIBL-IFOAM Report. Geneva; Bonn; Frick: IFOAM; FiBL; ITC.

[B67] Williams C. M. (2002). Nutritional quality of organic food: shades of grey or shades of green? Proc. Nutr. Soc. 61, 19–24. 10.1079/PNS2001126 12002790

